# Association of rs2072183 SNP and serum lipid levels in the Mulao and Han populations

**DOI:** 10.1186/1476-511X-11-61

**Published:** 2012-05-30

**Authors:** Lin Miao, Rui-Xing Yin, Xi-Jiang Hu, Dong-Feng Wu, Xiao-Li Cao, Qing Li, Ting-Ting Yan, Lynn Htet Htet Aung, Jin-Zhen Wu, Wei-Xiong Lin

**Affiliations:** 1 Department of Cardiology, Institute of Cardiovascular Diseases, the First Affiliated Hospital, Guangxi Medical University, 22 Shuangyong Road, Nanning, 530021, Guangxi, People’s Republic of China; 2 Department of Molecular Biology, Medical Scientific Research Center, Guangxi Medical University, 22 Shuangyong Road, Nanning, 530021, Guangxi, People’s Republic of China

## Abstract

**Background:**

Niemann-pick C1-like 1 (NPC1L1) is a key protein for intestinal cholesterol transportation. Common single nucleotide polymorphisms (SNPs) in the NPC1L1 gene have been associated with cholesterol absorption and serum lipid levels. The present study was undertaken to explore the possible association of NPC1L1 rs2072183 1735 C > G SNP and several environmental factors with serum lipid levels in the Mulao and Han populations.

**Methods:**

Genotyping of the rs2072183 SNP was performed in 688 subjects of Mulao and 738 participants of Han Chinese. The interactions between NPC1L1 1735 C > G polymorphism and several environmental factors on serum lipid phenotypes were tested using the factorial design covariance analysis after controlling for potential confounders.

**Results:**

The frequency of G allele was lower in Mulao than in Han (29.72% *vs*. 37.26%, *P* < 0.001). The frequency of CC, CG and GG genotypes was 49.85%, 40.84% and 9.31% in Mulao, and 39.30%, 46.88% and 13.82% in Han (*P* < 0.001); respectively. The levels of low-density lipoprotein cholesterol (LDL-C), apolipoprotein (Apo) B and the ratio of ApoAI/ApoB in Han but not in Mulao were different among the three genotypes (*P* < 0.05 for all), the subjects with GG and CG genotypes had higher LDL-C, ApoB levels and lower ApoAI/ApoB ratio than the subjects with CC genotype. Subgroup analysis showed that the G allele carriers in Han had higher total cholesterol (TC), LDL-C and ApoB levels in males (*P* < 0.05) and lower ApoAI/ApoB ratio in both sexes (*P* < 0.05) than the G allele noncarriers. The G allele carriers in Mulao had higher TC and LDL-C levels in males (*P* < 0.05) and lower high-density lipoprotein cholesterol (HDL-C) levels in both sexes (*P* < 0.05) than the G allele noncarriers. Serum TC, LDL-C, ApoB levels and ApoAI/ApoB ratio were correlated with genotypes in Han males (*P* < 0.05) but not in females. Serum lipid parameters were also correlated with several environmental factors. The genotypes of rs2072183 SNP were interacted with gender or cigarette smoking to influence serum TC and HDL-C levels in Mulao, whereas the genotypes of rs2072183 SNP were interacted with several environmental factors to influence all seven lipid traits in Han (*P* < 0.05-0.01).

**Conclusions:**

The present study suggests that the rs2072183 SNP in NPC1L1 gene and its association with serum lipid profiles are different between the Mulao and Han populations. The difference in serum lipid profiles between the two ethnic groups might partly result from different rs2072183 SNP or NPC1L1 gene-environmental interactions.

## Introduction

The increased incidence of cardiovascular disease (CVD) in our today world has been linked to dyslipidemia. Unfavorable lipid profiles include high levels of plasma or serum total cholesterol (TC) [1,2], low-density lipoprotein cholesterol (LDL-C) [3,4] and apolipoprotein (Apo) B [5,6], and low levels of high-density lipoprotein cholesterol (HDL-C) and ApoAI [7,8]. Especially, elevated TC and LDL-C levels were well-established risk factors for atherosclerosis and coronary heart disease [2,9,10]. However, the source of the serum lipids in human mainly relies on endogenous synthesis and the intestinal absorption which occupied more than 50% of the amount from dietary [11]. Interestingly, the fraction intestinal cholesterol absorption ranges from 29-80% [12,13] and dietary influence on the plasma cholesterol and LDL-C levels is extremely variable among individuals [14]. Family history and twin studies have shown that genetic polymorphism could account for 40-60% of the interindividual variation in plasma lipid phenotypes [15-17]. Thus, these variations above to the cholesterol homeostasis suggest that some polymorphisms in the cholesterol absorption-related genes potentially affect the blood lipid levels [18-20].

The Niemann-Pick type C1-like 1 (NPC1L1) protein was identified as a cholesterol transporter and promoted the absorption of cholesterol and plant sterol from the intestinal lumen [21-24]. Moreover, it was the molecular target of ezetimibe which was a cholesterol absorption inhibitor verified to decrease TC and LDL-C levels [21,25]. NPC1L1 is a glycosylated protein localized at the brush-border membrane of the enterocyte [26]. The protein has the typical features of a membrane bound protein with a signal peptide sequence, 13 predicted transmembrane domains, and extensive N-linked glycosylation sites in the extracellular loop [27]. It also contains a sterol-sensing domain encompassing ~180 amino acids [11] and plays a critical role in cholesterol dependent regulation. The expression of NPC1L1 in intestine parallels the efficiency of cholesterol absorption along the gastrocolic axis, with the highest level of NPC1L1 expression and cholesterol absorption observed in the proximal jejunum and minimal NPC1L1 expression and cholesterol absorption in the ileum [27]. Furthermore, there is a small amount of expressions in other tissues such as liver, stomach, gallbladder and testis [19]. Some animal studies have shown that NPC1L1-deficient (NPC1L1−/−) mice were given high cholesterol diet; however, these mice did not appear hypercholesterolemia phenomenon caused by high cholesterol diet, instead exhibited approximate 70% substantial reduction in cholesterol absorption [20,21,23]. *In vitro* study, cholesterol intake reduced by 30% in NPC1L1 knockout CaCo-2 epithelial cells compare to a control group of normal CaCo-2 cells [28], and sterol uptake significantly increased by NPC1L1 overexpressing CaCo-2 epithelial cells in culture medium [23]. The human NPC1L1 gene, located on chromosome 7p13, includes 20 exons and 19 introns. It has been demonstrated that several variants in the NPC1L1 gene associated with cholesterol absorption and the blood lipid levels accordingly [14,20,29-31]. Several single nucleotide polymorphisms (SNPs) at the NPC1L1 gene in human have been shown to affect LDL-C lowing response to ezetimibe [32,33]. The 1735 C > G of rs2072183 SNP is in exon 2 and embedded in the coding regions. At present, many studies have focused on the NPCILl SNPs and their haplotypes associated with lipid levels sensitivity of the change which caused by the treatment with ezetimibe. But the association between rs2072183 SNP and blood lipid disorders and coronary heart disease is limited.

China has a vast territory and 56 ethnic groups. Han is the dominant ethnicity, and Mulao is a native minority existing 207,352 people according to the fifth national census statistics of China in 2000. A previous study has shown that the genetic relationship between Mulao and other minorities in Guangxi was much closer than that between Mulao and Han or Uighur nationality [34]. The associations of GALNT2, NCAN/CILP2/PBX4 and TRIB1 SNPs and serum lipid levels have been reported in our previous studies in this population [35-37]. However, information on the association of NPC1L1 SNPs and serum lipid profiles in the Mulao and Han populations has not been reported previously. Thus, the aim of the present study was to detect the association of NPC1L1 rs2072183 1735 C > G SNP and several environmental factors with serum lipid parameters in the Mulao and Han populations.

## Materials and methods

### Study population

The study population included 688 subjects of Mulao and 738 participants of Han Chinese who reside in Luocheng Mulao Autonomous County, Guangxi Zhuang Autonomous Region, People’s Republic of China. For the subjects of Mulao, there were 288 (41.86%) males and 400 (58.14%) females. The age ranged from 15 to 80 years, with an average age of 52.13 ± 14.16 years. For the participants of Han, there were 274 men (37.13%) and 464 women (62.87%). The age ranged from 15 to 80 years, with an average age of 52.13 ± 14.16 years. All of the subjects were randomly selected from our stratified randomized cluster samples. All of them were rural agricultural workers. No significant evidence indicated that they suffer from any chronic illness, including hepatic, renal, or thyroid. We excluded the subjects who had a history of heart attack or myocardial infarction, stroke, congestive heart failure, diabetes or fasting blood glucose ≥ 7.0 mmol/L determined by glucose meter. Subjects were not allowed to take agents of fibrates, statins, and hormones which impact on lipid metabolism or absorption. All subjects provided informed consent and the present study was approved by the Ethics Committee of the First Affiliated Hospital, Guangxi Medical University.

### Epidemiological survey

The survey was carried out using internationally standardized criteria, following a common protocol [38]. Information on demographics, socioeconomic status, and lifestyle factors was collected with standardized questionnaires. The alcohol information included questions about the number of liangs (about 50 g) of rice wine, corn wine, rum, beer, or liquor consumed during the preceding 12 months. Alcohol consumption was divided into three gradations: nondrinker (0), < 25 and ≥ 25 grams per day. Smoking status was also divided into three gradations: nonsmoker (0), < 20 and ≥ 20 cigarettes per day. At the physical examination, several parameters, such as height, weight, and waist circumference were measured. Body weight, to the nearest 50 grams, was measured using a portable balance scale. Subjects were weighed without shoes and in a minimum of clothing. Height was measured, to the nearest 0.5 cm, using a portable steel measuring device. Body mass index (BMI) was calculated as weigh in kg divided by the square of height in meters (kg/m^2^). Sitting blood pressure was measured three times with the use of a mercury sphygmomanometer after the subjects had a 5-minute rest, and the average of the three measurements was used for the level of blood pressure. Systolic blood pressure was determined by the first Korotkoff sound, and diastolic blood pressure by the fifth Korotkoff sound.

### Biochemical analysis

A venous blood sample of 5 mL was obtained from all subjects after an overnight fast. A part of the sample (2 mL) was collected into glass tubes and allowed to clot at room temperature, and used to measure serum lipid levels. Another part of the sample (3 mL) was transferred to tubes with anticoagulate solution (4.80 g/L citric acid, 14.70 g/L glucose, and 13.20 g/L tri-sodium citrate) and used to extract DNA. Immediately following clotting serum was separated by centrifugation for 15 minutes at 3000 rpm. The levels of TC, triglyceride (TG), HDL-C, and LDL-C in samples were determined by enzymatic methods with commercially available kits, Tcho-1, TG-LH (RANDOX Laboratories Ltd., Ardmore, Diamond Road, Crumlin Co. Antrim, United Kingdom, BT29 4QY), Cholestest N HDL, and Cholestest LDL (Daiichi Pure Chemicals Co., Ltd., Tokyo, Japan); respectively. Serum ApoAI and ApoB levels were detected by the immunoturbidimetric immunoassay using a commercial kit (RANDOX Laboratories Ltd.). All determinations were performed with an autoanalyzer (Type 7170A; Hitachi Ltd., Tokyo, Japan) in the Clinical Science Experiment Center of the First Affiliated Hospital, Guangxi Medical University.

### DNA amplification and genotyping

Genomic DNA was extracted from peripheral blood leukocytes using the phenol-chloroform method [39-42]. The extracted DNA was stored at 4 °C for the next experiment. The analyses of rs2072183 1735 C > G (trivial name L272L) SNP were performed by polymerase chain reaction and restriction fragment length polymorphism (PCR-RFLP). We amplified a 437 bp fragment using primers 5'-GGGATGACAGATAGCACCAA-3' (forward) and 5'-GACATCACCTTCCACCTCTTG-3' (reverse) (Sangon, Shanghai, People’s Republic of China). Each amplification reaction was performed using 100 ng genomic DNA in 25 μL of reaction mixture consisting of 1.0 μL of each primer (10 μmol/L), 12.5 μL 2 × *Taq* PCR MasterMix (constituent: 0.1 U *Taq* polymerase/μL, 500 μM dNTP each and PCR buffer;Tiangen, Beijing, People’s Republic of China) and 8.5 μL of ddH_2_O (DNase/RNase-free). The reaction of PCR begin as denaturizing at 95 °C for 5 min, then followed by 30 cycles of 30 s denaturation at 94 °C, 30 s annealing at 60 °C and extension 30 s at 72 °C, and the third stage was a final 8 min extension at 72 °C. After electrophoresis on a 2.0% agarose gel with 0.5 μg/mL ethidium bromide, the PCR products were visualized under ultraviolet light. Then 6 μL PCR amplifications were digested with 5 U *Taq* I restriction enzyme (Ferment) at 65 °C for 12 hours. After restriction enzyme digestion of the amplified DNA, genotypes were identified by electrophoresis on 2.0% agarose gels and visualized with ethidium-bromide staining ultraviolet illumination. The genotypes were scored by an experienced reader blinded to epidemiological data and serum lipid levels. Six samples (CC, CG and GG genotypes in two; respectively) detected by the PCR-RFLP were also confirmed by direct sequencing. The PCR products were purified by low melting point gel electrophoresis and phenol extraction, and then the DNA sequences were analyzed in Shanghai Sangon Biological Engineering Technology & Services Co., Ltd., People’s Republic of China.

### Diagnostic criteria

The normal values of serum TC, TG, HDL-C, LDL-C, ApoAI, ApoB levels, and the ratio of ApoAI to ApoB in our Clinical Science Experiment Center were 3.10-5.17, 0.56-1.70, 0.91-1.81, 2.70-3.20 mmol/L, 1.00-1.78, 0.63-1.14 g/L, and 1.00-2.50; respectively. The individuals with TC > 5.17 mmol/L and/or TG > 1.70 mmol/L were defined as hyperlipidemic [43,44]. Hypertension was diagnosed according to the criteria of 1999 World Health Organization-International Society of Hypertension Guidelines for the management of hypertension [45,46]. The diagnostic criteria of overweight and obesity were according to the Cooperative Meta-analysis Group of China Obesity Task Force. Normal weight, overweight and obesity were defined as a BMI < 24, 24–28, and > 28 kg/m^2^; respectively [47].

### Statistical analyses

Epidemiological data were recorded on a pre-designed form and managed with Excel software. Quantitative variables are expressed as mean ± standard deviation (serum TG levels are presented as medians and interquartile ranges). Qualitative variables are expressed as percentages. Allele frequency was estimated by gene counting, and the standard goodness-of-fit test was used to test the Hardy-Weinberg equilibrium. Chi-square test was used to compare the difference in genotype distribution between the populations. The difference in general characteristics between Mulao and Han was tested by the Student’s unpaired *t*-test. The association of genotypes and serum lipid parameters was tested by covariance analysis (with sex, age, BMI, blood pressure, wrist circumference, blood glucose, alcohol consumption, cigarette smoking as covariates). In order to evaluate the association of serum lipid levels with genotypes (CC = 1, CG = 2, GG = 3) and several environmental factors, multiple linear regression analysis with forward stepwise modeling was also performed in the combined population of Mulao and Han, Mulao, Han; respectively. Potential interactions between NPC1L1 1735 C > G polymorphism and several environmental factors on serum lipid phenotypes were tested using the factorial design covariance analysis after controlling for potential confounders in Mulao and Han; respectively. All reported *P* values were from two-sided tests and less than 0.05 was considered to be statistically significant. All statistical analyses performed using software package SPSS 13.0 (SPSS Inc., Chicago, Illinois).

## Results

### General characteristics and serum lipid levels

Table 1 gives the general characteristics and serum lipid levels between Mulao and Han populations. The levels of height, serum LDL-C, ApoB and the percentages of subjects who consumed heavy alcohol were higher in Mulao than in Han Chinese (*P* < 0.05-0.001), whereas the levels of BMI and diastolic blood pressure were lower in Mulao than in Han (*P* < 0.05). There were no significant differences in the levels of weight, wrist circumference, systolic blood pressure, pulse pressure, blood glucose, serum TC, TG, HDL-C, ApoAI, the ratio of ApoAI to ApoB, age structure, the percentages of subjects who smoked cigarettes, or the ratio of male to female between the two ethnic groups (*P* > 0.05 for all).

**Table 1:** Comparison of demography, lifestyle and serum lipid levels between the Mulao and Han populations

**Parameter**	**Han**	**Mulao**	***t*(**^**2**^**)**	***P***
Number	738	688	–	–
Male/female	274/464	288/400	3.340	0.068
Age (years)	51.95 ± 15.43	52.13 ± 14.16	−0.216	0.829
Height (cm)	154.11 ± 7.45	155.11 ± 8.21	−2.397	0.017
Weight (kg)	53.11 ± 8.72	53.03 ±9.51	0.177	0.859
Body mass index (kg/m^2^)	22.31 ± 2.98	21.97 ± 3.07	2.120	0.034
wrist circumference(cm)	75.17 ±7.77	75.29 ± 8.64	−0.276	0.783
Systolic blood pressure (mmHg)	129.95 ± 19.12	129.86 ± 21.90	−0.084	0.933
Diastolic blood pressure (mmHg)	82.40 ±10.96	81.15 ± 11.69	2.075	0.038
Pulse pressure (mmHg)	47.54 ± 14.55	48.70 ± 16.58	−1.398	0.164
Glucose	6.02 ± 1.65	6.00 ± 1.58	0.224	0.823
Cigarette smoking [n (%)]				
Nonsmoker	561(76.02)	518 (75.29)		
≤20 cigarettes/day	160(21.68)	141(20.49)		
>20 cigarettes/day	17 (2.30)	29 (4.22)	4.296	0.117
Alcohol consumption [n (%)]				
Nondrinker	601 (81.44)	519 (75.44)		
≤25 g/day	67 (9.08)	56 (8.14)		
>25 g/day	70 (9.48)	113(16.42)	15.357	0.000
Total cholesterol (mmol/L)	4.99 ± 1.10	5.07 ± 1.13	−1.297	0.195
Triglyceride (mmol/L)	1.07 (0.89)	1.08 (0.79)	−0.367	0.714
HDL-C (mmol/L)	1.73 ± 0.53	1.75 ± 0.43	−1.107	0.268
LDL-C (mmol/L)	2.88 ± 0.88	2.97 ± 0.88	−1.988	0.047
Apolipoprotein (Apo) AI (g/L)	1.33 ± 0.26	1.34 ± 0.39	−0.230	0.821
Apo B (g/L)	0.85 ± 0.21	0.99 ± 0.56	−6.312	0.000
Apo AI/Apo B	1.64 ± 0.49	1.58 ± 0.75	1.852	0.068

HDL-C, high-density lipoprotein cholesterol; LDL-C, low-density lipoprotein cholesterol. The value of triglyceride was presented as median (interquartile range), the difference between the two ethnic groups was determined by the Wilcoxon-Mann–Whitney test.

### Results of electrophoresis and genotyping

**Figure 1:**
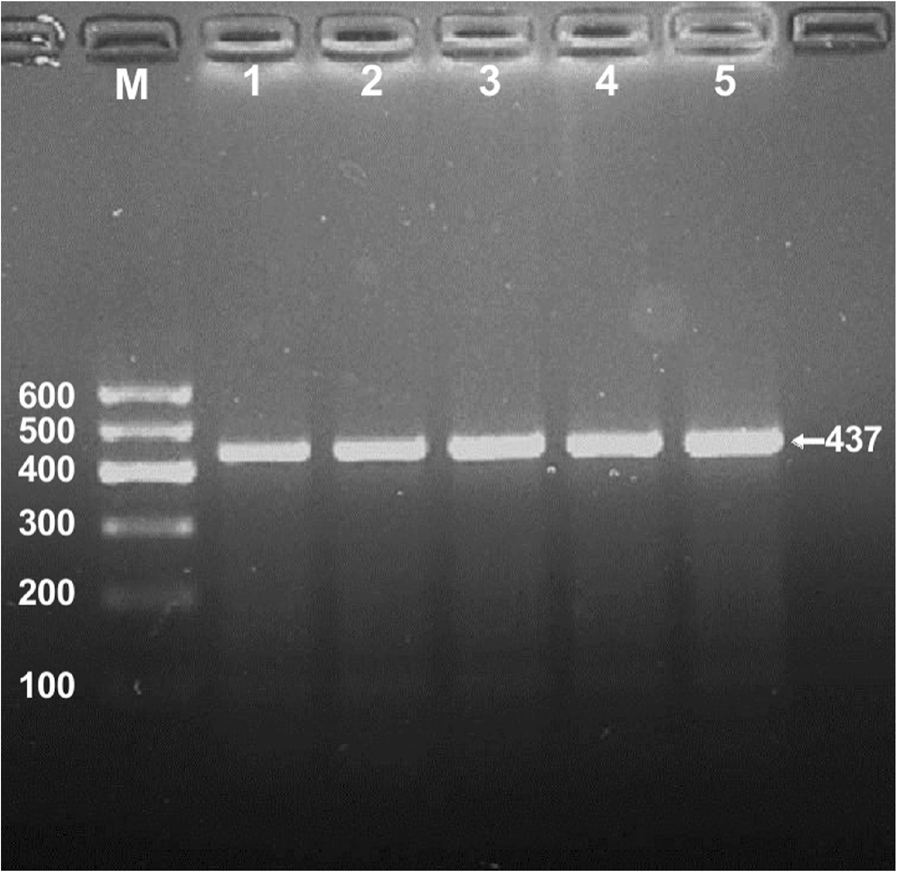
**Electrophoresis of PCR products of the samples.** Lane M, 100 bp marker ladder; lanes 1–5, samples. The 437 bp bands are the target genes.

**Figure 2:**
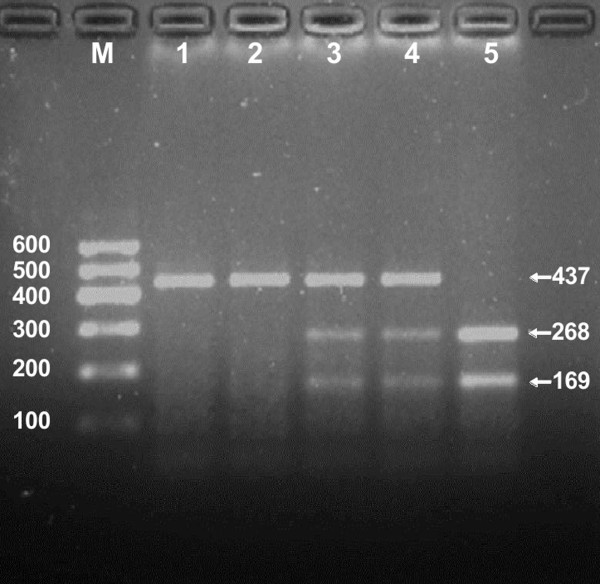
**Genotyping of the rs2072183 SNP.** Lane M, 100 bp marker ladder; lanes 1 and 2, CC genotype (437 bp); lanes 3 and 4, CG genotype (437-, 268- and 169-bp); and lane 5, GG genotype (268- and 169-bp).

After the genomic DNA of the samples was amplified by PCR and imaged by 2.0% agarose gel electrophoresis, the purpose gene of 437 bp nucleotide sequences could be found in all samples (Figure 1). The genotypes identified were named according to the presence or absence of the enzyme restriction sites, when a G to C transversion at 1735 locus of the NPC1L1 gene. The absence of the cutting site indicates the C allele; while its presence indicates the G allele (can be cut). Thus, the CC genotype is homozygote for the absence of the site (band at 437 bp), CG genotype is heterozygote for the absence and presence of the site (bands at 437-, 268- and 169-bp), and GG genotype is homozygote for the presence of the site (bands at 268- and 169-bp; Figure 2).

### Genotypic and allelic frequencies

The genotypic and allelic frequencies of rs2072183 SNP are shown in Table 2. The frequency of C and G alleles was 70.28% and 29.72% in Mulao, and 62.74% and 37.26% in Han (*P* < 0.001); respectively. The frequency of CC, CG and GG genotypes was 49.85%, 40.84% and 9.31% in Mulao, and 39.30%, 46.88% and 13.82% in Han (*P* < 0.001); respectively. There was no significant difference in the genotypic and allelic frequencies between males and females in both ethnic groups. The genotype distribution abides to the Hardy-Weinberg principle.

**Table 2:** Comparison of the genotype and allele frequencies of rs2072183 SNP in Mulao and Han Chinese [n (%)]

**Group**	**n**	**Genotype allelic**			**Allele**	
		**CC**	**CG**	**GG**	**C**	**G**
Han	738	290(39.30)	346(46.88)	102(13.82)	926(62.74)	550(37.26)
Mulao	688	343(49.85)	281 (40.84)	64 (9.31)	967(70.28)	409(29.72)
^2^	–	18.144			18.135	
*P*	–	0.000			0.000	
Han						
Male	274	100(36.50)	138(50.36)	36(13.14)	338(61.68)	210(38.32)
Female	464	190(40.95)	208 (44.83)	66(14.22)	588(63.36)	340(36.64)
*χ*^2^	–	2.142			0.418	
*P*	–	0.343			0.518	
Mulao						
Male	288	146 (50.70)	118(40.97)	24(8.33)	410(70.93)	168(29.07)
Female	400	197(49.25)	163(40.75)	40(10.00)	557(69.63)	243(30.37)
*χ*^2^	–	0.572			0.275	
*P*	–	0.751			0.600	

### Results of sequencing

**Figure 3:**
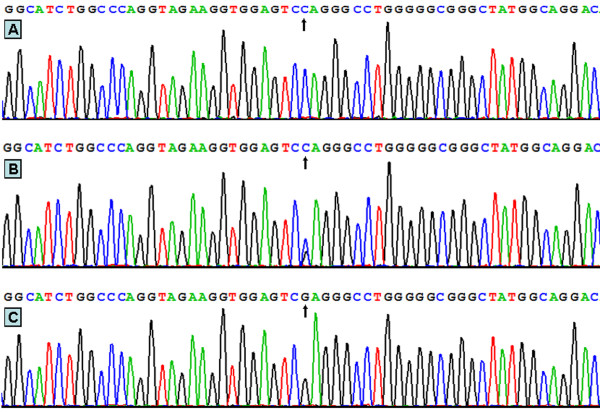
**A part of the nucleotide sequence of rs2072183 SNP.** (A) CC genotype; (B) CG genotype; (C) GG genotype.

The results were shown as CC, CG and GG genotypes by PCR-RFLP, the CC, CG and GG genotypes were also confirmed by sequencing (Figure 3); respectively.

### Genotypes and s**erum lipid levels**

As shown in Table 3, the levels of LDL-C, ApoB and the ratio of ApoAI to ApoB in Han but not in Mulao were different among the three genotypes (*P* < 0.05 for all), the subjects with GG and CG genotypes had higher LDL-C, ApoB levels and lower the ratio of ApoAI to ApoB than the subjects with CC genotype. When serum lipid levels were analyzed according to sex, the G allele carriers in Han had higher serum TC, LDL-C and ApoB levels in males (*P* < 0.05) and lower the ratio of ApoAI to ApoB in both sexes (*P* < 0.05) than the G allele noncarriers. The G allele carriers in Mulao had higher serum TC and LDL-C levels in males (*P* < 0.05) and lower HDL-C levels in both sexes (*P* < 0.05) than the G allele noncarriers. There was no significant difference in the remaining serum lipid parameters among the three genotypes in Mulao, Han, males, or females (*P* > 0.05 for all); respectively.

**Table 3:** Genotypes of the rs2072183 SNP and serum lipid levels between the Mulao and Han populations

**Genotype**	**n**	**TC (mmol/L)**	**TG (mmol/L)**	**HDL-C (mmol/L)**	**LDL-C (mmol/L)**	**ApoAI (g/L)**	**ApoB (g/L)**	**ApoAI/ApoB**
Han								
CC	290	4.90 ± 0.97	1.07(0.88)	1.70 ± 0.41	2.74 ± 0.77	1.34 ± 0.26	0.82 ± 0.20	1.72 ± 0.52
CG	346	5.05 ± 1.20	1.12(0.87)	1.74 ± 0.64	2.96 ± 0.92	1.33 ± 0.27	0.86 ± 0.21	1.59 ± 0.46
GG	102	5.14 ± 1.09	0.95(0.99)	1.75 ± 0.44	3.04 ± 0.97	1.32 ± 0.23	0.89 ± 0.22	1.59 ± 0.53
*F*	–	1.874	1.491	1.059	5.418	0.200	3.430	4.303
*P*	–	0.154	0.474	0.347	0.005	0.819	0.033	0.014
CC	290	4.90 ± 0.97	1.07(0.88)	1.70 ± 0.41	2.74 ± 0.77	1.34 ± 0.26	0.82 ± 0.20	1.72 ± 0.52
CG/GG	448	5.07 ± 1.18	1.06(0.89)	1.74 ± 0.60	2.98 ± 0.93	1.32 ± 0.26	0.87 ± 0.21	1.60 ± 0.47
*F*	–	3.650	−0.972	2.080	10.845	0.397	6.741	8.067
*P*	–	0.056	0.331	0.150	0.001	0.529	0.010	0.005
Male								
CC	100	5.06 ± 0.92	1.23(1.28)	1.61 ± 0.45	2.75 ± 0.77	1.36 ± 0.30	0.89 ± 0.20	1.59 ± 0.49
CG	138	5.35 ± 1.20	1.18(0.86)	1.73 ± 0.42	3.03 ± 0.86	1.38 ± 0.29	0.92 ± 0.21	1.57 ± 0.44
GG	36	5.39 ± 1.26	1.29(2.12)	1.56 ± 0.34	3.26 ± 0.92	1.24 ± 0.16	1.01 ± 0.23	1.28 ± 0.25
*F*	–	3.231	0.946	2.388	4.764	2.197	4.664	5.021
*P*	–	0.041	0.623	0.094	0.009	0.113	0.010	0.007
CC	100	5.01 ± 0.92	1.23(1.28)	1.61 ± 0.45	2.75 ± 0.77	1.36 ± 0.30	0.89 ± 0.20	1.59 ± 0.49
CG/GG	174	5.36 ± 1.20	1.22(0.92)	1.69 ± 0.41	3.08 ± 0.88	1.35 ± 0.27	0.94 ± 0.21	1.51 ± 0.42
*F*	–	6.460	−0.789	2.775	8.980	0.003	4.269	2.550
*P*	–	0.012	0.430	0.097	0.003	0.954	0.040	0.111
Female								
CC	190	4.81 ± 0.98	1.04(0.78)	1.75 ± 0.38	2.73 ± 0.77	1.33 ± 0.24	0.79 ± 0.19	1.79 ± 0.52
CG	208	4.84 ± 1.68	0.97(0.80)	1.75 ± 0.75	2.91 ± 0.96	1.29 ± 0.26	0.83 ± 0.20	1.62 ± 0.45
GG	66	5.01 ± 1.02	0.85(0.82)	1.85 ± 0.45	2.92 ± 0.97	1.37 ± 0.25	0.82 ± 0.18	1.77 ± 0.56
*F*	–	0.383	2.241	0.913	1.799	2.368	1.676	5.916
*P*	–	0.682	0.326	0.402	0.167	0.095	0.188	0.003
CC	190	4.81 ± 0.98	1.04(0.78)	1.75 ± 0.38	2.73 ± 0.77	1.33 ± 0.24	0.79 ± 0.19	1.79 ± 0.52
CG/GG	274	4.89 ± 1.13	0.96(0.80)	1.78 ± 0.68	2.91 ± 0.96	1.30 ± 0.26	0.83 ± 0.20	1.65 ± 0.49
*F*	–	0.201	−1.025	0.380	3.527	1.285	2.910	6.656
*P*	–	0.654	0.305	0.538	0.061	0.257	0.089	0.010
Mulao								
CC	343	5.08 ± 1.11	1.07(0.72)	1.76 ± 0.43	2.96 ± 0.87	1.35 ± 0.39	0.99 ± 0.56	1.60 ± 0.71
CG	280	5.10 ± 1.16	1.10(0.88)	1.75 ± 0.44	3.01 ± 0.93	1.32 ± 0.41	1.01 ± 0.60	1.56 ± 0.83
GG	64	4.90 ± 1.10	1.14(0.82)	1.75 ± 0.46	2.88 ± 0.77	1.33 ± 0.39	0.86 ± 0.27	1.63 ± 0.56
*F*	–	0.521	0.603	0.004	0.324	0.229	1.503	0.040
*P*	–	0.594	0.740	0.996	0.723	0.742	0.223	0.961
CC	343	5.08 ± 1.11	1.07(0.72)	1.76 ± 0.43	2.96 ± 0.87	1.35 ± 0.39	0.99 ± 0.56	1.60 ± 0.71
CG/GG	344	5.06 ± 1.15	1.10(0.86)	1.75 ± 0.44	2.99 ± 0.90	1.32 ± 0.40	0.98 ± 0.55	1.58 ± 0.79
*F*	–	0.112	−0.764	0.008	0.008	0.586	0.081	0.010
*P*	–	0.738	0.445	0.931	0.928	0.444	0.776	0.919
Male								
CC	146	5.05 ± 1.04	1.17(0.96)	1.71 ± 0.45	2.85 ± 0.75	1.39 ± 0.38	1.09 ± 0.68	1.54 ± 0.66
CG	118	5.34 ± 0.97	1.09(1.05)	1.83 ± 0.43	3.08 ± 0.85	1.36 ± 0.46	1.06 ± 0.61	1.49 ± 0.67
GG	24	4.89 ± 1.20	1.39(1.26)	1.73 ± 0.51	2.80 ± 0.71	1.36 ± 0.40	0.90 ± 0.32	1.62 ± 0.60
*F*	–	4.185	0.775	2.964	3.065	0.073	0.836	0.614
*P*	–	0.016	0.679	0.053	0.038	0.930	0.435	0.542
CC	146	5.05 ± 1.04	1.17(0.96)	1.71 ± 0.45	2.85 ± 0.75	1.39 ± 0.38	1.09 ± 0.68	1.54 ± 0.66
CG/GG	142	5.27 ± 1.02	1.12(1.02)	1.82 ± 0.45	3.03 ± 0.84	1.36 ± 0.45	1.03 ± 0.57	1.51 ± 0.66
*F*	–	3.075	−0.259	4.875	3.186	0.140	0.414	0.185
*P*	–	0.081	0.796	0.028	0.075	0.709	0.521	0.668
Female								
CC	197	5.09 ± 1.16	1.02(0.59)	1.80 ± 0.40	3.04 ± 0.94	1.32 ± 0.39	0.92 ± 0.43	1.63 ± 0.75
CG	162	4.92 ± 1.25	1.10(0.66)	1.68 ± 0.43	2.96 ± 0.98	1.29 ± 0.37	0.98 ± 0.59	1.62 ± 0.93
GG	40	4.91 ± 1.04	0.99(0.74)	1.76 ± 0.42	2.93 ± 0.80	1.30 ± 0.38	0.84 ± 0.24	1.64 ± 0.55
*F*	–	1.215	2.093	2.518	0.546	0.199	1.106	0.056
*P*	–	2.989	0.351	0.082	0.580	0.820	0.332	0.946
CC	197	5.09 ± 1.16	1.02(0.59)	1.80 ± 0.40	3.04 ± 0.94	1.32 ± 0.39	0.92 ± 0.43	1.63 ± 0.75
CG/GG	202	4.92 ± 1.22	1.09(0.68)	1.70 ± 0.43	2.95 ± 0.95	1.29 ± 0.37	0.95 ± 0.84	1.62 ± 0.87
*F*	–	2.247	−1.364	4.146	0.963	0.364	0.661	0.020
*P*	–	0.135	0.172	0.042	0.327	3.547	0.417	0.888

TC, total cholesterol; TG, triglycerides; HDL-C, high-density lipoprotein cholesterol; LDL-C, low-density lipoprotein cholesterol; ApoAI, apolipoprotein AI; ApoB, apolipoprotein B; ApoAI/ApoB, the ratio of apolipoprotein AI to apolipoprotein B. The value of TG was presented as median (interquartile range). The difference among the genotypes was determined by the Kruskal-Wallis test or the Wilcoxon-Mann–Whitney test.

### Relative factors for serum lipid parameters

Multiple linear regression analysis showed that serum LDL-C levels were correlated with genotypes in the combined population of Mulao and Han (*P* < 0.05). Serum LDL-C and ApoB levels were correlated with genotypes in Han (*P* < 0.05 for each) but not in Mulao (Table 4). When the association of rs2072183 genotypes and serum lipid levels were analyzed according to sex, we found that the levels of LDL-C, ApoB and the ratio of ApoAI to ApoB in Han were correlated with genotypes in males (*P* < 0.05 for all, Table 5) but not in females. Serum lipid parameters were also correlated with gender, age, BMI, wrist circumference, alcohol consumption, cigarette smoking, blood pressure and blood glucose in both ethnic groups (Table 4).

**Table 4:** Correlative factors for serum lipid parameters between the Mulao and Han populations

**Lipid parameter**	**Risk factor**	**Unstandardized coefficient**	**Std. error**	**Standardized coefficient**	***t***	***P***
Mulao plus Han						
TC	Wrist circumference	0.022	0.004	0.159	6.001	0.000
	Age	0.012	0.002	0.154	5.864	0.000
	Alcohol consumption	0.169	0.042	0.105	4.066	0.000
	Diastolic blood pressure	0.008	0.003	0.083	3.067	0.002
TG	Wrist circumference	0.055	0.006	0.245	9.379	0.000
	Alcohol consumption	0.240	0.078	0.090	3.089	0.002
	Blood glucose	0.098	0.030	0.086	3.309	0.001
	Cigarette smoking	0.302	0.103	0.084	2.930	0.003
	Diastolic blood pressure	0.011	0.004	0.069	2.600	0.009
	Age	−0.007	0.003	−0.056	−2.092	0.037
HDL-C	Wrist circumference	−0.008	0.002	−0.127	−3.261	0.001
	Alcohol consumption	0.120	0.022	0.170	5.419	0.000
	Age	0.002	0.001	0.07	2.732	0.006
	Gender	0.099	0.032	0.099	3.09	0.002
	Body mass index	−0.019	0.006	−0.116	−3.054	0.002
LDL-C	Age	0.011	0.002	0.187	7.331	0.000
	Wrist circumference	0.012	0.004	0.107	2.846	0.004
	Body mass index	0.029	0.011	0.099	2.638	0.008
	Ethnic group	0.12	0.046	0.068	2.635	0.009
	Alcohol consumption	−0.076	0.033	−0.06	−2.301	0.022
	Genotype	−0.073	0.034	−0.056	−2.179	0.030
ApoAI	Alcohol consumption	0.108	0.013	0.224	8.558	0.000
	Wrist circumference	−0.004	0.001	−0.089	−3.400	0.001
	Age	0.001	0.001	0.055	2.133	0.033
ApoB	Wrist circumference	0.010	0.001	0.185	7.025	0.000
	Ethnic group	0.133	0.021	0.159	6.287	0.000
	Blood glucose	0.024	0.007	0.093	3.638	0.000
	Gender	−0.068	0.022	−0.079	−3.042	0.002
	Systolic blood pressure	0.001	0.001	0.071	2.728	0.006
ApoAI/ApoB	Wrist circumference	−0.012	0.003	−0.150	−3.882	0.000
	Blood glucose	−0.030	0.010	−0.077	−2.919	0.004
	Age	−0.003	0.001	−0.070	−2.679	0.007
	Alcohol consumption	0.127	0.028	0.139	4.474	0.000
	Gender	0.177	0.041	0.137	4.333	0.000
	Body mass index	−0.023	0.008	−0.108	−2.867	0.004
	Ethnic group	−0.075	0.032	−0.059	−2.318	0.021
Mulao						
TC	Wrist circumference	0.022	0.005	0.170	4.538	0.000
	Age	0.011	0.003	0.132	3.525	0.000
TG	Wrist circumference	0.046	0.006	0.227	7.620	0.000
	Alcohol consumption	0.305	0.069	0.161	4.431	0.000
HDL-C	Body mass index	−0.022	0.007	−0.161	−3.045	0.002
	Alcohol consumption	0.094	0.021	0.163	4.462	0.000
	Wrist circumference	−0.008	0.003	−0.153	−2.890	0.004
	Age	0.003	0.001	0.095	2.609	0.009
LDL-C	Body mass index	0.052	0.011	0.183	4.911	0.000
	Age	0.009	0.002	0.144	3.865	0.000
	Alcohol consumption	−0.095	0.044	−0.081	−2.186	0.029
ApoAI	Alcohol consumption	0.107	0.020	0.202	5.344	0.000
	Wrist circumference	−0.003	0.002	−0.075	−1.982	0.048
ApoB	Wrist circumference	0.012	0.002	0.191	5.098	0.000
	Blood glucose	0.030	0.013	0.085	2.257	0.024
ApoAI/ApoB	Wrist circumference	−0.019	0.003	−0.218	−5.803	0.000
	Alcohol consumption	0.143	0.043	0.143	3.329	0.001
	Cigarette smoking	−0.163	0.060	−0.116	−2.726	0.007
	Blood glucose	−0.041	0.018	−0.086	−2.294	0.022
Han Chinese						
TC	Diastolic blood pressure	0.016	0.004	0.160	4.300	0.000
	wrist circumference	0.022	0.005	0.157	4.312	0.000
	Age	0.009	0.003	0.131	3.518	0.000
	Gender	−0.231	0.081	−0.101	−2.841	0.005
	Blood glucose	0.048	0.024	0.072	2.013	0.044
TG	Wrist circumference	0.092	0.015	0.330	6.132	0.000
	Gender	−0.422	0.161	−0.094	−2.627	0.009
	Blood glucose	0.146	0.047	0.111	3.083	0.002
	Diastolic blood pressure	0.025	0.007	0.125	3.357	0.001
	Age	−0.013	0.005	−0.092	−2.462	0.014
	Body mass index	−0.089	0.038	−0.123	−2.34	0.020
HDL-C	Wrist circumference	−0.012	0.003	−0.175	−4.834	0.000
LDL-C	Age	0.013	0.002	0.219	6.262	0.000
	Wrist circumference	0.021	0.004	0.188	5.364	0.000
	Genotype	−0.220	0.063	−0.122	−3.495	0.001
ApoAI	Body mass index	−0.012	0.003	−0.134	−3.660	0.000
	Gender	−0.045	0.020	−0.083	−2.266	0.024
ApoB	Wrist circumference	0.008	0.001	0.285	8.291	0.000
	Systolic blood pressure	0.001	0.000	0.080	1.798	0.073
	Blood glucose	0.021	0.004	0.165	5.008	0.000
	Gender	−0.062	0.015	−0.146	−4.246	0.000
	Genotype	−0.037	0.014	−0.086	−2.656	0.008
	Diastolic blood pressure	0.002	0.001	0.101	2.318	0.021
ApoAI/ApoB	Body mass index	−0.032	0.007	−0.197	−4.794	0.000

TC, total cholesterol; TG, triglyceride; HDL-C, high-density lipoprotein cholesterol; LDL-C, low-density lipoprotein cholesterol; ApoAI, apolipoprotein AI; ApoB, apolipoprotein B.

**Table 5:** Correlative factors for serum lipid parameters between males and females in both ethnic groups

**Lipid parameter**	**Risk factor**	**B**	**Std. Error**	**Beta**	**t**	**Sig.**
Han/male						
TC	Diastolic blood pressure	0.029	0.006	0.284	5.015	0.000
	Alcohol consumption	0.227	0.076	0.168	2.984	0.003
	Blood glucose	0.093	0.036	0.151	2.709	0.007
	Genotype	−0.302	0.129	−0.130	−2.346	0.020
	Wrist circumference	0.016	0.008	0.116	2.024	0.044
TG	Wrist circumference	0.115	0.022	0.298	5.174	0.000
	Cigarette smoking	0.934	0.304	0.177	3.073	0.002
HDL-C	Body mass index	−0.052	0.008	−0.371	−6.279	0.000
	Alcohol consumption	0.108	0.029	0.210	3.695	0.000
	Diastolic blood pressure	0.006	0.002	0.164	2.809	0.005
LDL-C	Genotype	−0.218	0.076	−0.169	−2.87	0.005
	Cigarette smoking	−0.224	0.086	−0.155	−2.596	0.010
	Body mass index	0.038	0.017	0.134	2.276	0.024
ApoAI	Alcohol consumption	0.126	0.019	0.369	6.546	0.000
	Body mass index	−0.025	0.005	−0.270	−4.686	0.000
	Diastolic blood pressure	0.003	0.001	0.128	2.277	0.024
	Cigarette smoking	0.059	0.027	0.124	2.208	0.028
ApoB	Wrist circumference	0.008	0.001	0.294	5.399	0.000
	Diastolic blood pressure	0.004	0.001	0.228	4.230	0.000
	Blood glucose	0.022	0.006	0.184	3.450	0.001
	Alcohol consumption	0.037	0.013	0.148	2.753	0.006
	Genotype	−0.043	0.017	−0.137	−2.586	0.010
ApoAI/Apo B	Body mass index	−0.061	0.008	−0.412	−7.541	0.000
	Alcohol consumption	0.108	0.030	0.200	3.647	0.000
	Genotype	0.097	0.036	0.144	2.681	0.008
	Blood glucose	−0.030	0.014	−0.118	−2.185	0.030
Han/female						
TC	Alcohol consumption	−0.477	0.230	−0.091	−2.073	0.039
	Age	0.026	0.003	0.349	8.007	0.000
	Body mass index	0.065	0.016	0.178	4.089	0.000
TG	Wrist circumference	0.046	0.008	0.269	6.010	0.000
	Diastolic blood pressure	0.015	0.005	0.136	3.054	0.002
	Blood glucose	0.107	0.033	0.138	3.191	0.002
HDL-C	Wrist circumference	−0.009	0.004	−0.113	−2.445	0.015
LDL-C	Cigarette smoking	−0.559	0.232	−0.107	−2.410	0.016
	Age	0.022	0.003	0.352	7.891	0.000
	Wrist circumference	0.019	0.005	0.156	3.572	0.000
ApoAI	Body mass index	−0.008	0.004	−0.092	−2.009	0.045
	Cigarette smoking	0.188	0.067	0.129	2.796	0.005
ApoB	Wrist circumference	0.003	0.002	0.110	1.656	0.098
	Blood glucose	0.018	0.005	0.149	3.399	0.001
	Age	0.003	0.001	0.249	5.386	0.000
	Body mass index	0.012	0.004	0.182	2.788	0.006
	Cigarette smoking	−0.135	0.050	−0.119	−2.681	0.008
ApoAI/B	Body mass index	−0.040	0.008	−0.232	−5.354	0.000
	Age	−0.009	0.002	−0.246	−5.511	0.000
	Cigarette smoking	0.613	0.133	0.206	4.610	0.000
Mulao/male						
TC	Wrist circumference	0.022	0.007	0.192	3.314	0.001
TG	Wrist circumference	0.067	0.011	0.341	6.201	0.000
	Alcohol consumption	0.299	0.109	0.151	2.754	0.006
HDL-C	Alcohol consumption	0.118	0.027	0.235	4.333	0.000
	Wrist circumference	−0.015	0.003	−0.297	−5.464	0.000
	Age	0.005	0.002	0.151	2.787	0.006
LDL-C	Body mass index	0.036	0.015	0.140	2.399	0.017
ApoAI	Alcohol consumption	0.123	0.026	0.266	4.659	0.000
ApoB	Wrist circumference	0.009	0.004	0.134	2.294	0.023
ApoAI/Apo B	Alcohol consumption	0.162	0.041	0.222	3.959	0.000
	Wrist circumference	−0.017	0.004	−0.233	−4.150	0.000
Mulao/female						
TC	Age	0.015	0.004	0.190	3.897	0.000
	Body mass index	0.056	0.019	0.146	2.985	0.003
TG	Wrist circumference	0.029	0.007	0.209	4.311	0.000
	Alcohol consumption	1.028	0.283	0.176	3.639	0.000
HDL-C	Body mass index	−0.034	0.007	−0.250	−5.150	0.000
LDL-C	Body mass index	0.062	0.015	0.204	4.256	0.000
	Age	0.014	0.003	0.220	4.584	0.000
ApoB	Wrist circumference	0.014	0.003	0.221	4.553	0.000
	Blood glucose	0.037	0.018	0.104	2.095	0.037
	Age	0.003	0.002	0.100	2.027	0.043
ApoAI/Apo B	Wrist circumference	−0.019	0.005	−0.186	−3.820	0.000
	Age	−0.008	0.003	−0.150	−3.082	0.002

TC, total cholesterol; TG, triglycerides; HDL-C, high-density lipoprotein cholesterol; LDL-C, low-density lipoprotein cholesterol; Apo AI, Apo lipoprotein AI; Apo B, Apo lipoprotein B.

### Interaction between rs2072183 SNP and several environmental factors on serum lipid phenotypes

Interaction between rs2072183 genotypes and gender or cigarette smoking was shown to influence serum TC and HDL-C levels in Mulao, whereas interaction between rs2072183 SNP and several environmental factors were found to influence all seven lipid traits in Han (*P* < 0.05-0.01; Table 6).

**Table 6:** Interaction between rs2072183 SNP and several environmental factors on serum lipid phenotypes

**Environmental factor**	***F/P***	**TC**	**TG**	**HDL-C**	**LDL-C**	**ApoAI**	**ApoB**	**ApoAI/ApoB**
Han Chinese								
Age	*F*	1.685	0.768	1.830	0.492	1.730	0.867	1.410
	*P*	0.099	0.631	0.068	0.862	0.088	0.544	0.189
Gender	*F*	1.336	0.860	1.576	0.581	5.460	1.787	5.977
	*P*	0.263	0.424	0.207	0.559	0.004	0.168	0.003
Body mass index	*F*	2.406	4.276	4.111	3.110	2.370	6.915	6.440
	*P*	0.091	0.014	0.017	0.045	0.094	0.001	0.002
Wrist circumference	*F*	1.722	0.144	3.928	2.695	1.545	5.992	4.495
	*P*	0.179	0.886	0.020	0.068	0.214	0.003	0.011
Systolic blood pressure	*F*	0.637	0.016	0.927	1.194	0.107	0.337	0.496
	*P*	0.529	0.940	0.396	0.304	0.899	0.714	0.609
Diastolic blood pressure	*F*	2.618	0.268	0.297	5.737	0.238	3.047	1.409
	*P*	0.074	0.765	0.743	0.003	0.789	0.048	0.245
Blood glucose	*F*	0.549	0.038	0.120	4.463	0.048	1.396	1.682
	*P*	0.578	0.963	0.887	0.012	0.954	0.248	0.187
Alcohol consumption	*F*	4.880	2.770	0.157	1.135	2.142	2.066	1.004
	*P*	0.001	0.026	0.960	0.339	0.074	0.084	0.404
Cigarette smoking	*F*	1.864	2.810	1.422	2.229	0.460	0.276	0.053
	*P*	0.115	0.025	0.225	0.064	0.765	0.893	0.995
Mulao								
Age	*F*	0.511	1.450	0.592	1.034	1.402	0.647	0.548
	*P*	0.849	0.172	0.785	0.408	0.192	0.739	0.821
Gender	*F*	3.461	0.283	7.360	1.681	0.021	1.019	0.084
	*P*	0.032	0.753	0.001	0.187	0.979	0.362	0.919
Body mass index	*F*	1.422	0.989	0.031	1.599	0.439	0.056	0.086
	*P*	0.242	0.372	0.969	0.203	0.645	0.945	0.918
Wrist circumference	*F*	1.656	2.006	0.231	0.606	0.465	0.132	0.049
	*P*	0.192	0.135	0.794	0.546	0.628	0.876	0.952
Systolic blood pressure	*F*	2.098	0.751	0.514	1.460	1.060	0.921	1.156
	*P*	0.124	0.472	0.598	0.233	0.347	0.399	0.315
Diastolic blood pressure	*F*	0.937	0.836	0.080	0.165	1.023	0.074	1.591
	*P*	0.392	0.434	0.923	0.848	0.360	0.929	0.204
Blood glucose	*F*	2.041	0.566	2.071	0.628	0.395	1.960	2.200
	*P*	0.131	0.568	0.127	0.534	0.674	0.142	0.112
Alcohol consumption	*F*	0.830	1.000	1.437	0.629	0.396	0.479	0.228
	*P*	0.506	0.407	0.220	0.642	0.811	0.751	0.923
Cigarette smoking	*F*	2.429	0.604	2.900	1.124	1.559	0.831	0.548
	*P*	0.047	0.660	0.021	0.344	0.183	0.505	0.700

Environmental factors were scored as follow: BMI (≤ 24 kg/m^2^ = 1, > 24 kg/m^2^ = 2); Wrist circumference (≤ 80 cm = 1, > 80 cm = 2); Systolic blood pressure (< 140 mmHg = 1, ≥ 140 mmHg = 2); Diastolic blood pressure (< 90 mmHg = 1, ≥ 90 mmHg = 2); Blood glucose (≤ 6.1 mmol/L = 1, > 6.1 mmol/L = 2); Alcohol consumption (nondrinker = 0, < 25 g/d = 1, ≥ 25 g/d = 2); Cigarette smoking (nonsmoker = 0, < 20 cigarettes/d = 1, ≥ 20 cigarettes/d = 2).

## Discussion

Our investigation revealed that the levels of serum LDL-C and ApoB were higher in Mulao than in Han Chinese. There was no significant difference in the levels of TC, TG, HDL-C, ApoAI and the ratio of ApoAI to ApoB between the two ethnic groups. It was widely realized that dyslipidemia as a serious risk factor for CVD caused by various elements, mainly including genetic and environmental factors and their interaction [41-44]. Concerning the customs of Mulao minority, the young were betrothed with the deliberation by both families when they were in childhood, usually with the girl being four or five years older than the boy. Usually the maternal female cousin was the priority target for marriage. The girl got married at an early age and remained with her natal family until her first child was born. Till then she was free to join the young men and women who came together for responsive singing, flirtations, and courtships at festival times. Divorce and remarriage were permitted, with little restriction. The two-generation household is the most common unit of residence. Households are under the control of the father, and divide when the sons marry, with only the youngest son remaining with the parents. Owing to its own unique marriage system, so we speculate that the genetic polymorphisms of the lipid-associated genes in Mulao may be different from those in the local Han residents.

The present study showed that the allelic and genotypic distribution of rs2072183 SNP was different in the Mulao and Han populations. The frequency of G allele was 29.72% in Mulao and 37.26% in Han (*P* < 0.001); respectively. The frequency of GG genotype in Han was also higher than that in Mulao (13.82% *vs*. 9.31%). There was no significant difference in the allelic and genotypic frequencies between males and females in the both ethnic groups. The frequency of minor allele (NPC1L1 1735 G) in our study was also different from that in several previous studies. Hegele *et al*. [33] reported that the frequency of G allele was 25.0% and the frequencies of CC and GG genotypes were 55% and 5% in 101 Canadian patients with primary hypercholesterolemia. They also found that there was moderate but not strong linkage disequilibrium (LD) among 1735 C > G, 27677 T > C and 25342A > C, the most common haplotype was defined as 1735 C-25342A-27677 T whose frequency was 61.9%. Furthermore, Siomon *et al*. [32] reported that the minor allele (G) frequency was 21.9%, 28.3% and 17.9% in healthy Caucasians, African American and Hispanics; respectively. Among Asian populations, Chen *et al*. [20] screened the promoter and coding regions of NPC1L1 gene for genetic polymorphisms from 50 Chinese Taiwanese, and revealed that the two common SNPs of 1735 C > G and -762 T > C were highly linked (D'value = 0.7459, *P* < 0.0001). The frequency of G allele was 35.7% and the frequencies of CC and GG genotypes were 39.29% and 10.71%; respectively. These results were similar to those of our Han population. The frequencies of G allele and GG genotype promulgated by Maeda *et al*. [30] were 41.90% and 19.01% in Japanese, and were higher than those in Han Chinese. These results indicate that the prevalence of 1735 G allele variants in the NPC1L1 gene may have an ethnic specificity.

Several previous studies reported that the rs2072183 SNP could cause significant change in serum TC, LDL-C and ApoB levels independently, but others did not find the correlations. An authoritative primary meta-analysis containing 46 participating studies in the world revealed that the mutation of rs2072183 had an important correlation with serum TC and LDL-C levels [48]. Polisecki *et al*. [29] have demonstrated that this SNP was associated with slightly higher TC, LDL-C, and ApoB in elder European males and females, with 1735GG carriers had higher LDL-C levels compared with CC and CG carriers. However, Siomon *et al*. [32] reported that this SNP was not related to basal cholesterol concentration in 375 apparently healthy individuals which consist of 198 Caucasians, 99 African Americans and 78 Hispanics. Zhao *et al*. [24] also showed a similar result in 82 hypercholesterolemic men. Nevertheless, Zhao *et al*. found that rs2072183 SNP could increase the responsiveness to plant sterols. Heterozygous carriers demonstrated a trend of an enhanced cholesterol lowering effect in response to plant sterols intervention, as compared to homozygous counterparts [24]. On the contrary, the mutant G allele carriers (*n* = 37) showed a trend of a greater reduction in serum TC (−9.8 ± 2.0% *vs*. -4.1 ± 1.6%, *P* = 0.057) and LDL-C (−14.5 ± 3.3% *vs*. -4.4 ± 2.5%, *P* = 0.082) in comparison with their wild type counterparts (C/C, *n* = 42). Thus the polymorphisms could be useful in devising individualized cholesterol lowering strategies [24]. Likewise, Maeda *et al*. [30] found that the presence of G allele had no effect on serum TC, HDL-C, LDL-C and ApoB levels, there was no significant difference among the CC, CG and GG genotypes in 139 Japanese. But when makers of cholesterol synthesis/absorption were compared between the GG and CG/CC genotypes, the campesterol level was significantly higher in the GG than CG/CC genotypes, and the sitosterol level tended to be higher. Chen *et al*. [20] also reported the genetypic distribution of rs2072183 SNP did not differ significantly between serum cholesterol level < 24 and > 24 groups (*P* > 0.05) in Chinese Taiwanese. In addition, some haplotypes were related with the polymorphism site have been given especial concern. For instance, the variation company with two haplotype blocks (TaqSNP:-18A > C and U328650A > G) formed haplotypes. A test showed that carriers of common haplotype (−18A-1735 C- U328650A > G) had 1.8 mg/dl lower baseline LDL-C than other haplotypes [29]. These data manifested that the variation and haplotype were significantly associated with LDL-C levels, and the homozygotes carriers for the minor allele had the higher LDL-C level. The most frequent combination 1735 C-25342A-27677 T was defined as haplotype 2 and all the other haplotypes as haplotype X. Interestingly, all of Maeda *et al*. [30] and Hegele *et al*. [33] showed that the levels of serum TC, TG, HDL-C and LDL-C have no significant difference among these haplotypes. In the present study, we showed that the levels of LDL-C, ApoB and the ratio of ApoAI to ApoB in Han but not in Mulao were different among the three genotypes, the subjects with GG and CG genotypes had higher LDL-C, ApoB levels and lower the ratio of ApoAI to ApoB than the subjects with CC genotype. When serum lipid levels were analyzed according to sex, the G allele carriers in Han had higher serum TC, LDL-C and ApoB levels in males and lower the ratio of ApoAI to ApoB in both sexes than the G allele noncarriers. The G allele carriers in Mulao had higher serum TC and LDL-C levels in males and lower HDL-C levels in both sexes than the G allele noncarriers. The reason for these conflicting results is not fully understood, probably because of differences in study designs, sample size, race, the methods used to determine serum lipid levels and the effectiveness of this polymorphism or interaction with other SNPs, as well as gene-environmental interactions.

In addition to genetic polymorphism, serum lipid levels in our study populations were also affected by many environmental factors. Multiple linear regression analysis showed that serum lipid parameters were also affected by several environmental factors such as age, gender, BMI, wrist circumference, alcohol consumption, cigarette smoking, blood pressure and blood glucose. Although rice and corn are the staple foods in both ethnic groups, with the improvement of local living standards, the diet structure of Mulao has changed gradually. They eat too many high-cholesterol foods such as fat, animal offal, spinal cord and brain, which can directly raise blood cholesterol concentrations. They also like to eat fried foods containing a large number of trans-fatty acids which have been consistently shown in multiple and rigorous randomized trials to have adverse effects on blood lipids [49,50], thereby increase the probability of suffering from coronary heart disease [51-53]. In our study populations, the percentages of subjects who consumed heavy alcohol were higher in Mulao than in Han. It is well known that moderate ethanol intake could increases the level of HDL-C and decreased risk of CVD [54,55], but consumption of large amounts of ethanol, or binge drinking would have the opposite result [56]. Alcohol consumption also has an important influence on the level of LDL-C [57]. Perissinotto *et al*. [57] reported that alcohol intake increase serum LDL-C levels in older Italian subjects (65–84 years old). Another recent study of Turks also found increases in LDL-C, as well as in ApoB and TG, with alcohol in men, while women had decreased TG and no change in LDL-C or ApoB with alcohol [58]. In addition, a greater amount of alcohol intake was also associated with higher values for blood pressure [57,59], TG [58,60], HDL-C [55] and waist circumference [60].

It is well known that interactions between genetic and environmental factors play an important role in determining serum lipid levels [61,62]. In the present study, we detected the potential interactions between rs2072183 SNP and several environmental factors including age, gender, BMI, wrist circumference, systolic blood pressure, diastolic blood pressure, blood glucose, alcohol consumption, and cigarette smoking on serum lipid phenotypes. The results showed that the genotypes of rs2072183 SNP were interacted with gender or cigarette smoking to influence serum TC and HDL-C levels in Mulao, whereas the genotypes of rs2072183 SNP were interacted with several environmental factors to influence all seven lipid traits in Han. These results suggest that some environmental factors in our study populations might affect serum lipid levels directly and/or indirectly by gene-environmental interactions. Furthermore, the NPC1L1 gene-environmental interactions on serum lipid levels were different between the two ethnic groups. However, there are still many unmeasured environmental and genetic factors and their interactions in the present study. Thus, the interactions of environment-environment, environment-gene, and gene-gene on serum lipid levels remain to be determined.

## Conclusion

The present study shows that the association of NPC1L1 1735 C > G polymorphism and serum lipid levels is different between the Mulao and Han populations. The difference in the association of NPC1L1 1735 C > G polymorphism and serum lipid levels between the two ethnic groups might partly result from different NPC1L1 1735 C > G polymorphism and/or NPC1L1 gene-environmental interactions.

## Competing interests

The authors declare that they have no competing interests.

## Authors’ contributions

LM participated in the design, undertook genotyping, and drafted the manuscript. RXY conceived the study, participated in the design, carried out the epidemiological survey, collected the samples, and helped to draft the manuscript. XJH, DFW, XLC, QL, TTY and LHHA collaborated to the genotyping. JZW and WXL carried out the epidemiological survey, collected the samples, and helped to carry out the genotyping. All authors read and approved the final manuscript.
